# Manufacturing of a Secretoneurin Drug Delivery System with Self-Assembled Protamine Nanoparticles by Titration

**DOI:** 10.1371/journal.pone.0164149

**Published:** 2016-11-09

**Authors:** Bernhard Scheicher, Cornelia Lorenzer, Katrin Gegenbauer, Julia Partlic, Fritz Andreae, Alexander H. Kirsch, Alexander R. Rosenkranz, Oliver Werzer, Andreas Zimmer

**Affiliations:** 1 Department of Pharmaceutical Technology, Institute of Pharmaceutical Sciences, University of Graz, Graz, Austria; 2 piCHEM, Kahngasse 20, Graz, Austria; 3 Department of Internal Medicine, Clinical Division of Nephrology, Medical University of Graz, Auenbruggerplatz 27, Graz, Austria; Universite du Quebec a Trois-Rivieres, CANADA

## Abstract

Since therapeutic peptides and oligonucleotides are gathering interests as active pharmaceutical ingredients (APIs), nanoparticulate drug delivery systems are becoming of great importance. Thereby, the possibility to design drug delivery systems according to the therapeutic needs of APIs enhances clinical implementation. Over the last years, the focus of our group was laid on protamine-oligonucleotide-nanoparticles (so called proticles), however, the possibility to modify the size, zeta potential or loading efficiencies was limited. Therefore, at the present study we integrated a stepwise addition of protamine (titration) into the formation process of proticles loaded with the angiogenic neuropeptide secretoneurin (SN). A particle size around 130 nm was determined when proticles were assembled by the commonly used protamine addition at once. Through application of the protamine titration process it was possible to modify and adjust the particle size between approx. 120 and 1200 nm (dependent on mass ratio) without influencing the SN loading capacity. Dynamic light scattering pointed out that the difference in particle size was most probably the result of a secondary aggregation. Initially-formed particles of early stages in the titration process aggregated towards bigger assemblies. Atomic-force-microscopy images also revealed differences in morphology along with different particle size. In contrast, the SN loading was only influenced by the applied mass ratio, where a slight saturation effect was observable. Up to 65% of deployed SN could be imbedded into the proticle matrix. An in-vivo biodistribution study (i.m.) showed a retarded distribution of SN from the site of injection after the application of a SN-proticle formulation. Further, it was demonstrated that SN loaded proticles can be successfully freeze-dried and resuspended afterwards. To conclude, the integration of the protamine titration process offers new possibilities for the formulation of proticles in order to address key parameters of drug delivery systems as size, API loading or modified drug release.

## Introduction

The formulation of an effective nanoparticulate drug delivery system requires consideration of various physicochemical properties. The therapeutic effect of an active pharmaceutical ingredient (API) is strongly influenced by the size and the surface charge of the delivery system [1, 2] as well as from the hydrophobicity [3] and morphology [4]. These properties are controlling the bioavailability of the API [5] as well as the release profile from the delivery system [3], and will further affect cellular uptake [1, 2] and targeting issues. Thus, the success in clinical implementation is based on sufficient API loading, but also on the possibility to design delivery systems according to the therapeutic needs of the API. However, the modification of physicochemical properties of delivery systems is often limited or they are influencing each other.

Over the last 15 years, the focus of our group was laid on the formulation of nanoparticulate drug delivery systems based on protamine and oligonucleotides (ODNs), so called proticles [6]. So far, the possibility to provide various proticle formulations differing in size and loading capacity was restricted to a formation method by self-assembly. To overcome these limitation, at our present study we describe a stepwise addition of protamine (titration) for the formulation of secretoneurin (SN) loaded proticles. SN-proticle formulations should further address a retarded release of SN from the delivery system in order to serve as depot formulation. Therefore, the integration of the protamine titration process also aimed to increase the size of the nanoparticles, as studies pointed out that more retarded release profiles are observed for larger particles [3]. A CpG-control ODN was used at all proticle formulations as negatively charged component for particle assembly, because our earlier studies already revealed that proticles consisting of CpG-control ODNs and protamine do not show immunostimulatory side effects at all [7].

Secretoneurin is a direct angiogenic cytokine consisting of 33 amino acids, which was described first by Kirchmair et al. in 1993 [8]. The active peptide is generated by proteolytic cleavage out of the precursor protein secretogranin II by prohormone convertase 1 and 2 [9]. Typically, SN is expressed in neuro-/ endocrine and neuronal tissues [8], but the expression of SN is up-regulated under ischemic conditions in skeletal muscle cells [10] and cardiac myocytes [11]. Several studies have demonstrated that SN has stimulative effects on neovascularization by induction of vasculogenesis, angiogenesis and arteriogenesis [12–14]. These pro-angiogenic effects are mainly based on proliferation of endothelial- and endothelial progenitor cells as well as on the stimulation of mitogenic and antiapoptotic processes. Further, the recruitment of pericytes and smooth muscle cells was observed [13, 14]. Intracellularly, SN activates the AKT (protein kinase B) and the mitogen activated protein kinase (MAPK) pathway [11, 12] and also stimulates the endothelial nitric oxide synthase [14]. Beside the effects on different cell types, SN shows upregulation and pathway stimulation of other angiogenic cytokines, like VEGF [11] and b-FGF [14]. Through its pro-angiogenic effects SN presents a promising drug candidate for the treatment of peripheral arterial diseases, ischemic heart disease and enhanced wound healing, which was already evaluated using methylcellulose patches [15].

Within the last years, several drug delivery systems were described for the application of angiogenic compounds. Thereby, different types of nanoparticles were investigated, e.g. protamine/heparin nanoparticles for delivery of FGF growth factor [16, 17] or coated gold nanoparticles either to activate or inhibit angiogenesis in vitro [18]. Nanoparticulate delivery systems are widely used because they can offer the protection of the API from rapid enzymatic degradation as well as the delivery of the bioactive peptide in respect of the integrity of its structure. These systems can further be equipped with targeting sequences in order to deliver APIs to the site of action, which is favored e.g. in cancer therapy to reduce side effects [19, 20]. Nanoparticulate depot formulations are also described in literature, which show retarded and sustained release kinetics [21].

Although there have been studies on SN gene therapy [14, 15, 22], a drug delivery system for the active peptide is not yet available. Therefore, proticles loaded with SN offer an attractive possibility for an intramuscular depot application to trigger prolonged and targeted angiogenesis with less frequent injections for the patient.

One of the major advantages of proticles is their simple formation process through a self-assembly process based on ODN condensation by the arginine rich protamine [6]. Therefore, neither extensive equipment nor harsh conditions as high pressure homogenization, ultrasound, or unfavorable pH values are necessary for particle formation. Proticles were already successfully applied as delivery system for different types of ODNs [7, 23], siRNA and thiomers. Peptides were initially added to proticle formulations in order to increase the stability under physiological conditions [24]. This aspect was further developed and proticles could already be used as drug delivery system for several peptides [25, 26]. Proticle research addressed issues like drug-targeting [27], vaccination [26] as well as depot-effects [25], and was recently reviewed in detail [28]. However, the only possibilities to vary the size of proticles were either to adapt the mass ratio or the concentration of the components. It was also shown that using protamine sulfate instead of protamine free base caused a drastic size reduction of particles and that modification of the ionic strength in solution can also have an effect on particle size [29]. Though, our studies also revealed that the mass ratio plays a crucial role in loading capacities of the components. Therefore, the possibilities of finding an optimal proticle formulation are limited.

At the present study, on the one hand we aimed to successfully imbed SN into the matrix of proticles in order to provide a delivery system for the peptide. Otherwise, we wanted to investigate if the dependency of mass ratio on particle size and surface charge can be minimized. Thus, the approach of a stepwise protamine titration process was introduced to achieve a more controlled proticle assembly and to offer new possibilities in the development of peptide loaded proticle formulations. To sum up, we succeeded the embedding of SN into proticles and were able to show that a stepwise protamine addition can modify the size of proticles without influencing the SN loading capacity of the delivery system. Results pointed out that the alteration in particle size is due to a secondary aggregation of initially-formed particles within the titration process.

## Material and Methods

### Materials

Secretoneurin (SN) and Alexa Fluor® 647 labeled SN were synthesized and purified by piChem (Graz, Austria) and provided as freeze-dried product. Full length SN sequence was used as reported by Fischer-Colbire et al: TNE IVE EQY TPQ SLA TLE SVF QEL GKL TGP NNQ [30]. Single stranded DNA oligonucleotides (ODN 1826 control—5’-TCC ATG AGC TTC CTG AGC TT-3’) were obtained from InvivoGen (San Diego, USA) and contain full phosphorothioate backbone. ODN 1826 control does not cause any immunostimulatory effect and was therefore used as non-active ODN component. Protamine free base (Grade IV, Histone free) was purchased from Sigma Aldrich (Vienna, Austria). For our in-vivo biodistribution study oligonucleotides were synthesized by Biospring (Frankfurt am Main, Germany) with the same sequence and backbone properties as mentioned above.

Stock solutions for each component were prepared in Milli-Q (MQ) water and stored at -24°C. MQ water (ultra-pure water) was provided by a Milli-Q-Gradient system (Merck-Millipore, Darmstadt, Germany). In addition, the water was sterilized before usage at 121°C for 20 min.

For HPLC measurements, acetonitrile (HPLC gradient grade) was obtained from VWR (Leuven, Belgium) and trifluoroacetic acid from Merck (Darmstadt, Germany). Mannitol was purchased from Carl Roth (Vienna, Austria) and was used as cryoprotectant for lyophilization.

### Particle formation

The formation of proticles occurs through a self-assembly process based on oligonucleotide condensation by the arginine rich protamine (up to 70% arginine residues [31]). Condensation and subsequent particle formation is induced by mixing the components in aqueous solution [6]. Before mixing, pre-dilutions of equal volumes were prepared separately in MQ water for each component (ODN, SN, protamine).

Initially, SN was added to the ODN dilution and mixed accurately. After 10 min incubation time at room temperature the protamine dilution was added at once or by a seven step titration process. Therefore, the protamine dilution was divided into 7 equal parts and added separately after an incubation time of 10 min. Pre-studies revealed that splitting the protamine solution in more than 7 aliquots will not change the properties of the resulting nanoparticles. When the protamine dilution was added by a single addition the particle characterization was done consequently after incubation of 10 min.

The concentration of ODNs in the final particle dispersion was set to 100 μg/ml throughout all proticle formulations. SN and protamine concentrations varied according to the declared mass ratios, given in consecutive order of addition as ODN:SN:protamine. SN varied between 25, 50, 75, and 100 μg/ml in the final particle dispersion and protamine was used in concentrations of either 150 or 300 μg/ml.

### Lyophilization of particles

The lyophilization of the nanoparticles was carried out using a Virtis Sentry freeze-drying unit and 1% (m/m) mannitol as cryoprotectant in order to achieve redispersability of the dried nanoparticles. Briefly, 150 μl of particle dispersion was transferred into 2R crimp vials (MGlas AG, Munnerstadt, Germany), followed by freezing in liquid nitrogen at atmospheric pressure and sublimation of water in vacuum below 6 mbar over 24 hrs. Freeze-dried particles were resuspended in 150 μl MQ water to achieve the same particle concentration as before the lyophilization process.

### Characterization of SN loaded particles

The mean particle size (hydrodynamic diameter, ZAve), the particle size distribution (polydispersity index, PdI) as well as the derived count rate (DCR) of particles were determined by dynamic light scattering (DLS), using a Zetasizer Nano ZS (Malvern Instruments, Malvern, UK) equipped with a green laser (532 nm). The size measurements were performed in a micro cuvette (Brand, Wertheim, Germany) by non-invasive back scatter mode (automatic mode), detecting the scattered light at 173°. The sample temperature was set to 25°C, and each measurement (including up to 20 sub-runs) was performed at least in duplicate. Results of the size measurements are always given as mean values of the hydrodynamic diameter in nanometer scale. To enable particle characterization throughout the titration process the DLS measurement was started 5 min after the protamine addition to achieve a total incubation time of 10 min between the several titration steps (see above).

Further, also the DCR of size measurements was evaluated. The DCR refers to the total intensity of scattered light without attenuation of the light source. As the attenuation factor of the measurement system is included in the DCR, it is calculated from the measured count rate at the detector and the applied attenuation factor [32], shown in equation 1:

$$DCR=measured\;count\;rate\times {attenuation\;factor}^{-1}.$$

For zeta potential measurements the samples were diluted 1:5 in MQ water and transferred into a clear folded capillary cell (Malvern Instruments, Malvern, UK). Primarily, the electrophoretic mobility of the particles was determined by the Zetasizer using Laser Doppler Velocimetry. Afterwards, the zeta potential was calculated from the electrophoretic mobility applying the Henry equation [33]. For all measurements the temperature was set to 25°C and the Helmholtz-Smoluchowski-Approximation was used for the f(Ka) value [33].

### Drug Loading and particle composition

Secretoneurin and protamine loading efficiencies were determined by an indirect quantification method using reversed phase high performance liquid chromatography (RP-HPLC). The particle dispersions were centrifuged immediately after the DLS measurements at 20817 rcf, at 4°C for 2 hrs (Eppendorf centrifuge 5804 R, Hamburg, Germany). Subsequently, the supernatants were transferred and centrifuged again for 2 hrs. SN and protamine concentration in the obtained supernatants were determined using external standards. For each HPLC series at least four different SN and protamine concentrations were prepared as external standards. Thereby, for each concentration two samples were prepared independently and injected twice (at the beginning and at the end of the series). The mean values of the four measurements were used in the calibration line for further determination.

As shown in Eq 2, the loading efficiencies (LE) were calculated from the totally applied concentration of the component (C_A_) and the determined unbound concentration in the supernatant (C_S_).

$$LE=\left(1-(\frac{C_{S}}{C_{A}})\right)*100$$ Equation 2

Our inert HPLC system (Merck Hitachi) was equipped with a D-7000 Interface, L7200 autosampler, L-7120 pump and L-7300 column oven. The column temperature was set to 30°C using a Zorbax Extend C18 column for separation (4.6x100 mm, 3.5 μm—Agilent Technologies). The elution of the compounds was carried out by a water/acetonitrile gradient (96.5:3.5 to 33.5:66.5 within 15 min) with a constant flow rate of 1 ml/min. To avoid peak tailing 0.075% (v/v) trifluoroacetic acid was added to the mobile phase. Detection was carried out at 215 nm using an L-7400 UV-detector.

HPLC pre-tests pointed out that SN was partly remaining in the system at higher concentrations, and eluted with the following injection (carry-over). The same behavior was observed with a modified HPLC method reaching 98% acetonitrile at the end of the gradient. However, carry-over was especially observed when no or low concentrations of protamine were present in the measured sample. Therefore, all samples as well as the external standards were spiked with 75 μg/ml protamine to avoid undesired carry-over of SN and to achieve equal sample preparation. The positive effect of the protamine addition is most probably referred to an ion-pair formation of the positively charged protamine and negatively charged SN. However, the mixture of 75 μg/ml protamine and different SN concentrations did not show turbidity or aggregation, and the single peptides were well-separated by the applied method.

### Atomic force microscopy imaging (AFM)

Before the AFM measurements the particle dispersions were diluted 1:10 in MQ-water in order to enable the detection of single nanoparticles. The diluted dispersions were dropped on a silicon wafer (1 μl, 5 μl, 10 μl) and dried overnight at room temperature. To guarantee suitable wafer coating AFM test measurements were performed. Consequently, the wafer was dried again for 2 days under vacuum at room temperature to remove remaining water.

AFM images were recorded using a Nanosurf AFM with an Easyscan 2 controller (Nanosurf, Liestal, Switzerland). All measurements were performed at non-contact mode with a Tap190Al-G cantilever (Budgetsensors, Romania). Further, image analysis and determination of particle dimensions was accomplished using Imaris 7.3.0 (Bitplane, Zürich, Switzerland) and compared to results from DLS measurements.

### In vivo biodistribution scan

For the in vivo biodistribution scan SN was labeled with fluorescent dye Alexa Fluor® 647 and applied either in solution or assembled into the proticle matrix. Alexa Fluor® 647 is known for unusually low fluorescence quenching upon protein conjugation [34], and was therefore used as fluorescent dye. Both SN formulations were injected intramuscularly (i.m., 30 μl) into hind limb of partly shaved C57BL/6 mice (Charles River, Sulzfeld, Germany—n = 8, euthanasia was not applicable). Animals were scanned under anesthesia at different times by optical in vivo imaging (Maestro™2.2, In vivo Fluorescence Imaging System, Cri, Inc., Woburn, MA, US). Pictures from the mouse (recorded under day light) and the fluorescent signal were stored separately and subsequently merged with Imaris 7.3.0. Representative results of the formulations are shown in the results part. All animal procedures were approved by the Austrian Federal Ministry of Science, Research and Economy (BMFWF).

### Statistical analysis

Hydrodynamic diameters from proticles assembled by different preparation methods were analyzed by one-way ANOVA (analysis of variance) to verify statistical significance. F-test was performed whether equal or unequal variances had to be assumed followed by a student’s t-test (two-tailed) to compare mean values. A p value < 0.05 was considered to be statistically significant different.

## Results

### Characterization of proticles

The characterization of the SN-proticle formulations focused on the mean particle size (hydrodynamic diameter, ZAve d.nm), the particle size distribution (polydispersity index, PdI) and SN loading. Various mass ratios were applied by adding protamine in a single step or by a stepwise titration process in order to verify the effect of the manufacturing process and the mass ratio on size and loading efficiencies.

**Fig 1:**
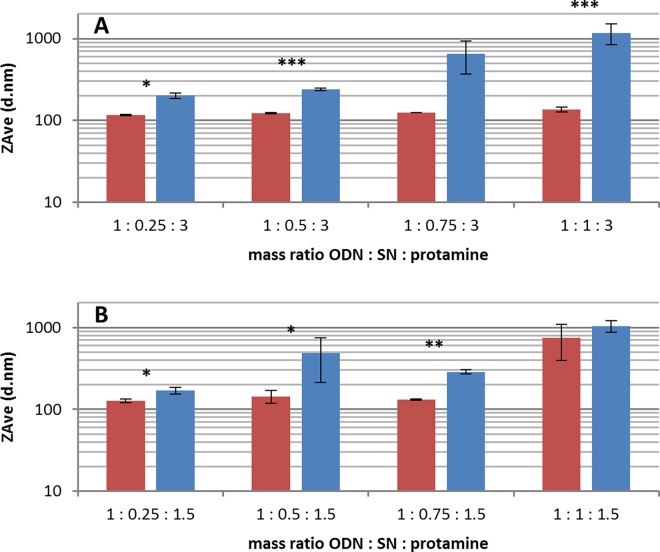
Mean particle size of secretoneurin loaded proticles obtained by different mass ratios and preparation methods. The size of particles is given as mean hydrodynamic diameter (ZAve d.nm) separated by the applied preparation method (red bars: single protamine addition, blue bars: titration process) and mass ratio (1 = 100 μg/ml). (A) shows particles with 300 μg/ml protamine and (B) with 150 μg/ml. The results are given as mean values +/- standard deviation, each sample was measured at least in duplicate (number of samples are listed in Table 1). Statistical significance between preparation methods is marked as * (p<0.05), ** (p<0.01) and *** (p<0.001).

Mean hydrodynamic diameters around 120 nm were recorded when 300 μg/ml protamine was applied by a single addition (Fig 1A, red bars). When the protamine titration process was used, higher ZAve values were recorded at all mass ratios, more evident at higher SN concentrations (Fig 1A, blue bars). Thereby, particles with 50 μg/ml SN showed ZAve values of 240 ± 7 nm, which pointed out a particle size around two-fold higher than after single protamine addition. When 75 μg/ml and 100 μg/ml SN were applied, a secondary aggregation was observed during the titration process (described below), reaching much larger agglomerated nanoparticles with 1179 ± 329 nm at the mass ratio 1:1:3 (ODN:SN:protamine). At the mass ratio 1:0.75:3 not all samples aggregated towards bigger assemblies, which resulted in a high standard deviation.

As shown in Fig 1B, particles with 150 μg/ml protamine generally showed the same influence. The protamine titration process resulted in higher ZAve values than protamine addition at once. At the mass ratio 1:0.25:1.5 only a moderate increase in the hydrodynamic diameter was observed (127 ± 6 nm to 170 ± 17 nm). Applying 75 μg/ml SN, the titration process again led to a particle size which was found to be two-fold higher than after a single protamine addition (287± 18 nm respectively 131 ± 2 nm). Larger agglomerated particles were also observed during the titration process, but only at the highest SN concentration of 100 μg/ml. When the same mass ratio was assembled by a single protamine addition, ZAve values of 902 ± 70 nm were obtained, which is in contrast to all other mass ratios. Usually, a particle size lower than 150 nm was determined after protamine was added at once. At the mass ratio 1:0.5:1.5 a very large deviation of particle size was recorded after the protamine titration. ZAve values were ranging from 369 nm up to 926 nm, which indicated that this mass ratio is not suitable for protamine titration.

In DLS measurements, the particle size distribution is generally expressed as polydispersity index. PdI values of SN loaded particles are summarized in Table 1. Particles with 150 μg/ml protamine showed no difference between the two preparation methods. A narrow size distribution was obtained, indicated by PdI values < 0.2. Similar results were recorded for particles with 300 μg/ml protamine after a single protamine addition. However, when the titration process was used for particle assembling, higher PdI values were determined. Thereby, a tendency towards a broader particle size distribution was observed at higher SN concentrations.

**Table 1:** PdI values of secretoneurin loaded proticles.

Mass ratio (ODN: SN)	300 μg/ml protamine		150 μg/ml protamine	
	titration process	single addition	titration process	single addition
1: 0.25	0.19 ±0.04 ^4^	0.19 ± 0.00 ^4^	0.12 ± 0.00 ^4^	0.18 ± 0.03 ^2^
1: 0.5	0.28 ± 0.02 ^4^	0.20 ± 0.01 ^4^	0.19 ± 0.06 ^2^	0.11 ± 0.07 ^3^
1: 0.75	0.27 ± 0.10 ^4^	0.20 ± 0.01 ^4^	0.12 ± 0.02 ^4^	0.18 ± 0.01 ^4^
1: 1	0.40 ± 0.10 ^2^	0.23 ± 0.03 ^3^	0.15 ± 0.06 ^1^	0.10 ± 0.06 ^3^

PdI values are given as mean values +/- standard deviation. Each sample was measured at least in duplicate

^1^ n = 12

^2^ n = 6

^3^ n = 5

^4^ n = 3

The zeta potential (ZP) was measured for formulations with 25 μg/ml and 100 μg/ml SN assembled by single protamine addition to cover the highest and lowest SN concentration. Our nanoparticles showed ZPs higher than +40 mV pointing out a highly positive surface charge. For comparison of the different preparation methods, ZPs were also determined for particles with 100 μg/ml SN assembled by protamine titration. Thereby, even slightly higher ZP values were recorded.

Proticles without SN were prepared in order to investigate the self-assembly and especially the titration process regardless of a third component. Applying 150 μg/ml protamine, no significant difference in size was obtained, whether protamine was added by a titration process (224 ± 5 nm) or a single addition (233 ± 2 nm). However, formulations with 300 μg/ml protamine showed a different performance. Now, a single protamine addition resulted into smaller nanoparticles with a ZAve of 140 ± 4 nm, whereas particles prepared by titration showed much higher values of 476 ± 103 nm. Recorded PdI values around 0.2 were similar to particles with SN, but after titration of 300 μg/ml protamine higher values of 0.47 ± 0.06 were observed.

Generally, particle formation only occurred when protamine and ODN were present in the formulation. A mixture of 100 μg/ml SN and 300 μg/ml protamine did not result in turbidity and showed a low DCR of 1,183 ± 592 kcps. Similar values were recorded for mixtures of ODN and SN, which are presented in later sections.

### Loading efficiencies

**Fig 2:**
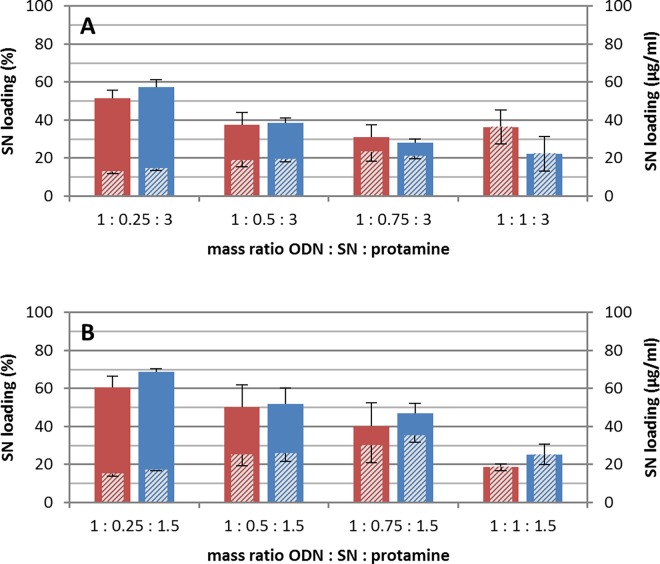
Secretoneurin (SN) loading of proticles obtained by different mass ratios and preparation methods. Secretoneurin (SN) loading is shown as relative percentage of applied SN and as absolute concentration (shaded bars). Red bars are referred to particles prepared by a single protamine addition and blue bars to the titration process. (A) shows the formulations with 300 μg/ml protamine and (B) with 150 μg/ml. The results are given as mean values incl. standard deviation, each sample was measured in duplicate (number of samples are listed in Table 1).

In general, the preparation method showed no influence on the SN loading of the proticles. In contrast to the particle size measurements mentioned above, all mass ratios showed similar loading efficiencies whether protamine was added at once or by a stepwise titration process (Fig 2). Further, our results pointed out that SN loading was mainly influenced by the mass ratio of the components.

The highest SN loading efficiency was determined at the mass ratio of 1:0.25:1.5 (ODN:SN:protamine). Using the titration process 69 ± 1.5% of SN was imbedded into the particle matrix and 61 ± 6% when protamine was added in a single step (Fig 2B). However, at higher SN concentrations successive lower loading efficiencies were obtained. These results indicated a saturation effect, thus, the more SN was applied the lower loading efficiency was determined. Consequently, at a mass ratio of 1:1:1.5 only 25 ± 5% of SN was incorporated by the titration process and 19 ± 2% after single protamine addition.

The same influence of SN concentration was observed for particles with 300 μg/ml protamine (Fig 2A), however, slightly less SN was incorporated compared to a protamine concentration of 150 μg/ml.

The evaluation of the protamine loading efficiency pointed out that around 35% of the deployed protamine was incorporated at formulations with 300 μg/ml protamine. With a protamine concentration of 150 μg/ml, a protamine loading around 76% was determined. This again indicated a saturation of peptide loading, which is comparable to SN. Remarkably, the protamine loading into the proticles was influenced by neither the assembly method nor the SN concentration. Even without SN a similar amount of protamine was assembled when 150 μg/ml were applied.

### Protamine titration process

**Fig 3:**
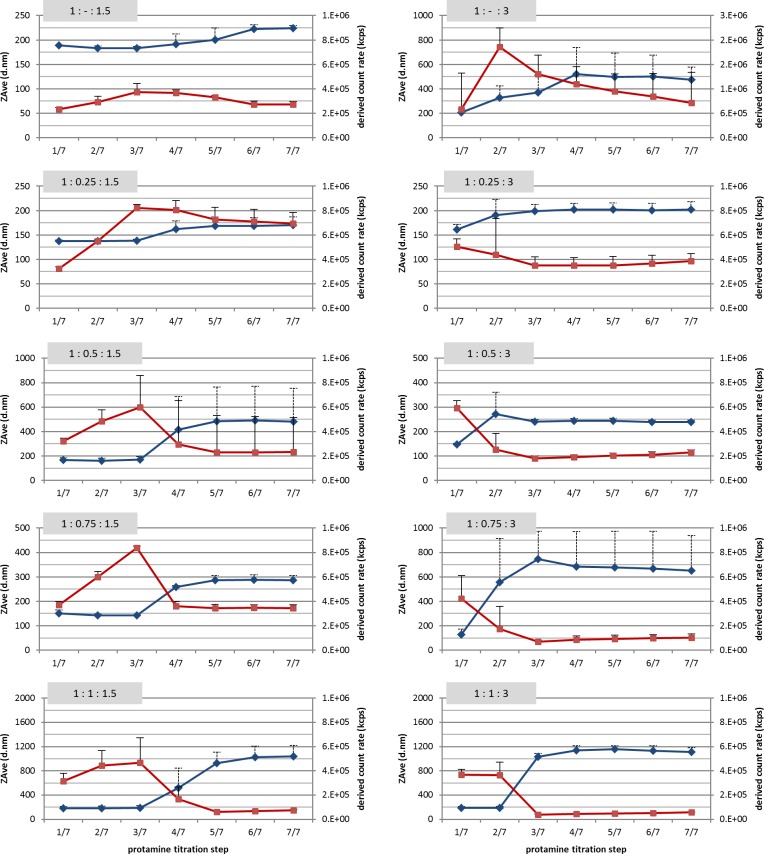
Correlation of mean particle size and derived count rate throughout protamine titration process. Development of mean particle size (ZAve, blue lines) and derived count rate (red lines) for each mass ratio (captioned on the top left of each graph). Horizontal axis indicates the steps of protamine addition along the titration process. The results are given as mean values + standard deviation (dashed error bars refer to particle size).

As already shown above, the stepwise titration process resulted in larger mean particle sizes compared to a single protamine addition. Therefore, the progression of the particle size along with consecutive titration steps was investigated in detail. Our results indicated a correlation between the mean particle size and the derived count rate (DCR) of DLS measurements (Fig 3). As the DCR refers to the total scattering intensity of a DLS measurement without attenuation, it can be used as a measure for the particle concentration in dispersion, which was already applied in other studies [32, 35].

In our study we determined the nanoparticle assembly (visualized by turbidity) already after the first titration step, regardless of protamine or SN concentration. The initial mixture of ODN and SN did not result in any kind of nanoparticulate formation, indicated by a lack of turbidity and a very low DCR (see Table 2). Further, no difference in the DCR could be observed between the mass ratios. When a nanoparticulate formation would occur, different values should be observed due to different SN concentrations. After the first protamine titration step a significant increase in DCR was observable at all formulations. Thereby, a higher DCR was recorded for formulations with 300 μg/ml protamine than with 150 μg/ml protamine. Concerning particle size, no differences in hydrodynamic diameter were obtained after the first protamine addition. ZAve values around 155 nm were recorded throughout all mass ratios.

**Table 2:** Derived count rate (DCR) of the initial ODN/SN mixtures.

Mass ratio ODN: SN: Prot	DCR (kcps)	
	before protamine addition	after first protamine addition
1: 0.25: 1.5	1,522 ± 530	322,960 ± 15,240
1: 0.5: 1.5	1,511 ± 399	319,684 ± 25,309
1: 0.75: 1.5	1,402 ± 87	369,804 ± 28,774
1: 1: 1.5	3,294 ± 2,553	316,772 ± 62,688
1: 0.25: 3	1,685 ± 350	505,697 ± 62,501
1: 0.5: 3	1,544 ± 179	592,246 ± 59,567
1: 0.75: 3	2,058 ± 426	423,413 ± 185,910
1: 1: 3	1,794 ± 1,036	369,094 ± 42,899

The DCR of initial ODN/SN mixtures (before protamine addition) is compared to values obtained after the first titration step (after first protamine addition). DCR is presented as mean value ± standard deviation.

By titration of 150 μg/ml protamine in total, the particle size remained constant within the first three steps (Fig 3 left column). Then, an increase of the mean particle size was observable, which was more evident at higher SN concentrations. Also the following titration step provoked a slight increase in particle size, but then remained constant until the end of the titration process. Only at mass ratio 1:1:1.5 also a weak increase after the sixth step was obtained. In contrast, the DCR steadily increased during the first steps of the titration, but along with particle growth a decrease of the DCR was determined. The observed decrease in DCR again was more pronounced when higher SN concentrations were applied. This indicated a secondary aggregation of pre-assembled nanoparticles, which were assembled at the very early titration steps.

The same correlation was observed during the titration of 300 μg/ml protamine (Fig 3 right column). However, the particle growth and the simultaneous decrease of DCR occurred earlier in the titration process, already at the second protamine addition. Only the mass ratio of 1:1:3 showed no difference in size and DCR between the first two titration steps. Subsequently, the mean particle size and the DCR were consistent after the third titration step. The decrease in DCR again was more evident at higher SN concentrations, but generally higher than at equal mass ratios with 150 μg/ml protamine.

**Fig 4:**
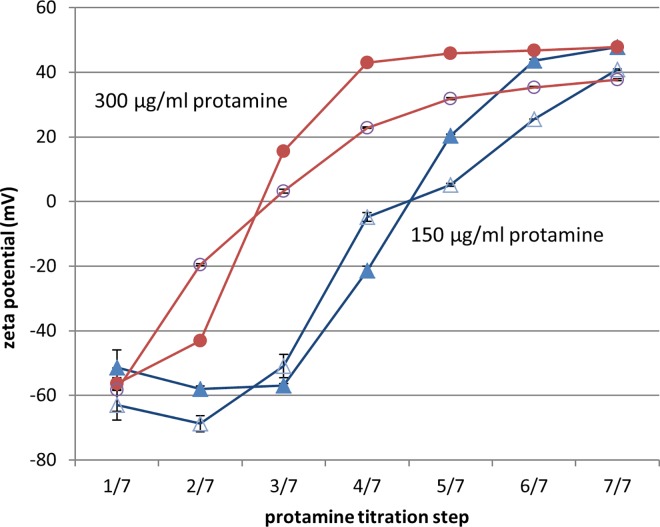
Development of zeta potential throughout protamine titration. Particles were assembled without SN (blank marks) or 100 μg/ml SN (filled marks), as well as with 150 μg/ml protamine (blue lines) or 300 μg/ml (red lines). The ODN concentration was set to 100 μg/ml, each undiluted sample was measured at least in duplicate.

Zeta potential measurements showed that the assembled nanoparticles at the first titration step are indicated by a highly negative ZP around -60 mV (Fig 4). These findings were regardless of the applied protamine concentration and whether 100 μg/ml SN were used for particle formation or no SN was applied. Throughout the titration process, the ZP changed from negative to neutral charge at the same titration step as particle size increased and DCR decreased. By further protamine titration, the ZP reached positive values and remained constant until the end of the titration process (Fig 4, red lines). As the development of the ZP is comparable for proticles with 100 μg/ml SN and without SN, particles with other SN mass ratios should behave similarly. This is also supported by the consistent correlation of ZAve and DCR throughout all mass ratios.

The obtained ZP distribution profiles are present in S1 Fig and revealed a single fraction for each measurement. This fraction could be related to the assembled nanoparticles, as the nanoparticles generally gain a higher scattering intensity than unbound peptides. Therefore, the average ZP will most probably be calculated from these nanoparticles and free peptides will not be determined.

### Lyophilization

The results of the DLS measurements after resuspension pointed out that SN loaded proticles can be lyophilized and successfully resuspended (Table 3). The mass ratio of 1:1:1.5 (ODN:SN:protamine) assembled by protamine titration was chosen as particle formulation with observed secondary aggregation. Furthermore, mass ratios 1:0.25:1.5 and 1:0.25:3 manufactured by a single protamine addition were tested as formulations with the highest SN loading capacity.

**Table 3:** DLS measurements of SN loaded particles before and after lyophilization.

Mass ratio—preparation method	before lyophilization		after lyophilization	
	ZAve (d.nm)	PdI	ZAve (d.nm)	PdI
1: 1: 1.5—titration process ^1^	1027 ± 97	0.18 ± 0.03	423 ± 78	0.5 ± 0.05
1: 0.25: 1.5—single addition ^2^	128 ± 6	0.15 ± 0.03	206 ± 25	0.23 ± 0.04
1: 0.25: 3—single addition ^3^	123 ± 1	0.18 ± 0.01	171 ± 22	0.20 ± 0.02

The mean particle size (ZAve d.nm) and the polydispersity index (PdI) of particles are shown before and after lyophilization. Each sample was measured in duplicate

^1^ n = 9

^2^ n = 6

^3^ n = 2

At all formulations, the resuspension of the nanoparticles in MQ-water did not show any kind of flocculation. ZAve values of particles with mass ratio of 1:1:1.5 pointed out that larger assemblies caused by secondary aggregation are partly disaggregating during the lyophilization process (Table 3). Higher PdI values also indicated a broader size distribution of particles after resuspension. On the other hand, initially smaller particles around 125 nm, obtained after single protamine addition, showed a higher mean particle size after resuspension.

### AFM imaging

**Fig 5:**
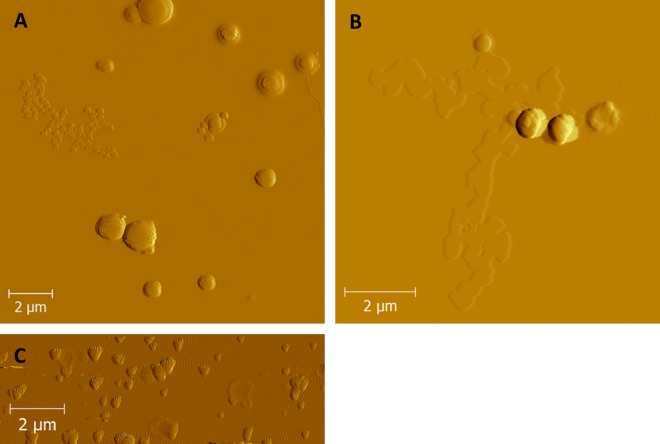
AFM images of secretoneurin loaded proticles. Images recorded by atomic force microscopy showed spherical secretoneurin loaded proticles for mass ratio of 1 : 1 : 1.5 prepared by titration process (A) as well as by single protamine addition (B). Triangular clustered proticles were recorded for mass ratio 1 : 0.25 : 1.5 prepared by protamine addition at once (C).

AFM images of proticles with mass ratio 1:1:1.5 resulted in round spherical particles at both preparation methods (Fig 5A and 5B). These findings are comparable to SEM (scanning electron microscope) images of proticles reported by Wernig et al. [25]. As the hydrodynamic diameter for both formulations was similar, it can be assumed that the preparation method has no influence on the particle shape. On the other hand, particles with mass ratio 1:0.25:1.5 assembled by single protamine addition showed triangular clustered structures (Fig 5C). These results pointed out that the particle shape is most probably influenced by the size of the assembled particles. The obtained particle dimensions were determined and are comparable to the results of the DLS measurements (S2 Fig).

At several spots on the silicon wafer a partial unwrapping of the nanoparticles occurred at mass ratio 1:1:1.5 after single protamine addition (Fig 5B). AFM images from the single components pointed out that these structures can be related to ODNs. As ODNs as well as the silicon wafers are negatively charged, electrostatic interactions are unlikely to provoke unwrapping. It’s more likely an artefact from the drying process, which was already reported in former proticle AFM measurements [36].

### In vivo biodistribution scan

After the application of fluorescently labeled SN (i.m.) the biodistribution of the peptide was observed in vivo. Therefore, SN was applied in solution as well as assembled within proticles. The proticle formulation with mass ratio 1:0.25:1.5 prepared by protamine single addition was chosen because of the highest SN loading efficiency. Therefore, only a low amount of unbound SN should be present and misinterpretation of non-assembled signal emitting peptides was minimized. Because of the low injection volume for i.m. administration in mice, the concentration of SN was set to 50 μg/ml in order to generate a sufficient fluorescent signal (ODN and protamine was adjusted according to given mass ratio). These particles showed ZAve values of 657 ± 78 nm with a narrow size distribution, indicated by PdI of 0.05 ± 0.02. Basically higher ZAve values were recorded compared to particles without the fluorescent dye. This difference in particle size can be the result of the employed fluorescent dye, but also from the higher concentration of the components. However, the difference is only less important for the obtained results of the biodistribution scan, because SN in solution is generally compared to a nanoparticulate formulation and not to a certain particle size. For each picture a second mouse was used as blank signal to monitor possible auto-fluorescence.

**Fig 6:**
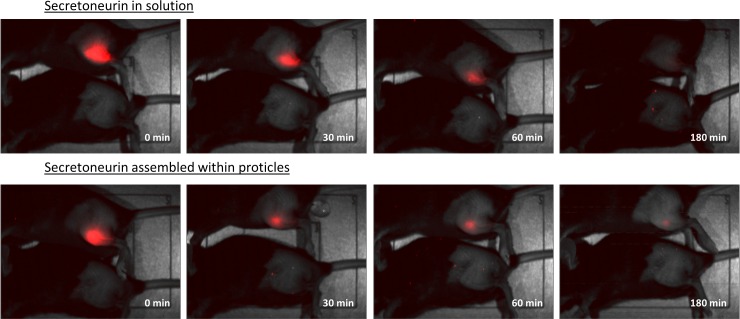
In-vivo biodistribution scan of fluorescently labeled secretoneurin in solution and assembled within proticles. Biodistribution of SN after intramuscular injection (30 μl) into hind limb of partly shaved C57BL/6 mice. Fluorescently labeled SN was applied in solution (50 μg/ml, upper panels) and assembled within the proticle matrix (mass ratio 1:0.25:1.5, lower panels).

The biodistribution scan pointed out that SN was remaining longer at the site of injection when assembled within proticles, whereas the application of SN in solution resulted in a more diffusive pattern (Fig 6, 30 min and 60 min). Further, 180 min after the injection a located signal was still detectable for the SN-proticle formulation, whereas SN in solution was highly distributed in the tissue, almost below the detection limit of the camera system.

## Discussion

The results from our study pointed out that the size of SN loaded proticles was mainly influenced by the preparation method, whereas the loading efficiencies of SN and protamine were only regulated by the mass ratio of the components. Thereby, a saturation effect for both peptides was obtained. However, the saturation effect of SN was not exactly linear, as the absolute concentration of assembled SN was slightly increasing with higher SN concentrations. The obtained saturation effect can be used to choose a desired ratio of incorporated and unbound amount of SN. The unbound amount can serve as initial dose of SN.

The saturation effect of the protamine loading was shown by loading efficiencies around 76% when 150 μg/ml were applied and 35% at protamine concentration of 300 μg/ml. This effect can be explained by the constant concentration of oligonucleotides throughout all formulations. The incorporation of protamine into the particle matrix was caused through binding to the negatively charged ODN backbone and further by the formation of an outer protamine layer surrounding the pre-formed particles. Therefore, with a constant ODN concentration the ability of protamine binding reached a maximum. Formulations with 150 μg/ml protamine seem to be more adequate for application, because of a lower amount of unbound protamine. Further, higher protamine concentrations did not result in different zeta potentials.

Concerning ODN binding, previous studies on proticles revealed that ODNs are completely assembled at a mass ratio of 1:1.25 (ODN:protamine) or higher, regardless of a phosphate or phosphorothioate backbone [23]. At present study we applied a 1.5 and 3 fold excess of protamine to ODN, so it can be assumed that for all mass ratios no unbound ODN was present. Complete ODN binding was further supported by a positive zeta potential of the resulting nanoparticles, which enabled sufficient SN loading. Preferentially, SN loading is more likely to positively charged particles, because SN is slightly negatively charged at neutral pH values, which is shown by an isoelectric point of 3.98 [37].

The difference in particle size between the two preparation methods was most probably due to secondary aggregation during the stepwise addition of protamine (titration). The correlation which was found between the hydrodynamic diameter and the derived count rate pointed out that the initially-formed particles within the first titration steps were aggregating towards bigger assemblies.

DLS measurements revealed that particles were assembled even at very low protamine concentration, also visualized by turbidity. The particle formation already occurred after the first protamine addition when 150 μg/ml protamine were added by titration, which corresponds to a mass ratio of 1:0.21 (ODN:protamine). This mass ratio pointed out that particle assembly was possible even at lower mass ratios than previously described [23, 38]. By further addition of protamine, neither the particle size nor the PdI has changed, but the increase in DCR indicated an increase in particle concentration. When the DCR is used to evaluate the particle concentration also size-related differences in scattering intensities [39] and the dilution through titration have to be considered. However, results of the DLS measurements showed that the size of the nanoparticles was remaining constant, therefore, an adverse effect by size-related differences in scattering intensities can be neglected. Consequently, higher DCR indicated the formation of new particles by continuous addition of protamine, with limited rearrangement of already existing particles.

In detail, not all ODN molecules were condensed into nanoparticles after the first titration step and were compacted by further protamine addition. This was also supported by findings of previous studies, where it was shown that unbound ODNs were present at a mass ratio of 1:0.5 (ODN:protamine). Further, zeta potential remained constantly negatively charged until secondary aggregation occurred. A negative surface charge of the nanoparticles is in line with previous studies on proticles assembled with an excess of ODN over protamine [23, 38]. During the titration process the zeta potential changed towards neutral values most likely due to binding of non-assembled protamine onto the surface of the initially-formed nanoparticles. A change in proticle surface charge due to protamine binding onto the surface was already discussed [40]. Microscopy images from Dinauer et al. also revealed a proticle structure with an inner protamine/ODN scaffold surrounded by an outer protamine layer [23]. As a result, secondary assemblies were formed due to reduced repulsion forces between the particles.

The formation of secondary assemblies was related to a calculated n/p (+/-) ratio of 1.48. Thereby, the molecular weight of the components was considered as well as 21 arginine residues per protamine molecule [31] and 18 phosphate groups of the ODN backbone. This ratio indicated that an excess of positive charge was necessary for complete neutralization of the ODN backbone and the formation of an outer protamine layer. Other groups also reported a correlation of +/- ratios close to unity and aggregation of polyelectrolyte complexes [41]. Thereby, also no difference was obtained whether the negatively or the positively charged compound was added.

In our study, the particle size of the secondary assemblies was mainly influenced by the applied SN and protamine concentrations. Higher concentrations of SN showed a more evident increase in particle size and protamine concentration of 300 μg/ml also resulted in a higher particle size than 150 μg/ml. In addition, this was also shown for a stepwise addition of protamine to ODN (without SN). The amount of unbound biomolecules probably regulated the growth of the secondary assemblies by acting as a linker between the initially-formed particles. The protamine titration process enabled the modification of the particle size through a more controlled proticle assembly. The protamine addition in a single step basically showed similar ZAve values at all mass ratios (except 1:1:1.5). However, the titration process showed a slight increase in particle size (170–280 nm), as well as intermediate (480–650 nm) and high particle sizes (> 1000 nm).

The secondary aggregation of pre-formed particles was also indicated by the extensive decrease in the DCR. As the DCR was compared between different particle sizes, scattering theories had to be considered. Generally, Mie theory describes the relation between the total scattering intensity and particle size, which is roughly equivalent to the wavelength of the light source. Thereby, the scattering intensity increases less with particle size (as it is known for small particles) and is also dependent on the applied wavelength, the scattering angle and the refractive index [39]. Mie theory shows that that total scattering intensity (DCR) is gradually increasing with particle size with strong fluctuations [42]. However, in our study the DCR was strongly decreasing along with secondary aggregation. Therefore, it can be supposed that the decrease in DCR also indicated a decrease in particle concentration.

Lyophilization studies pointed out that SN-proticle formulations showed both, an increase in mean particle size after resuspension, as well as smaller particles. Thereby, particles around 125 nm tend to increase in particle size after resuspension. This is in line with results from other research groups, which also showed that the particle size could be reduced by sonication afterwards [43, 44]. Sadeghi et al. also pointed out that only a few nanoparticles showed aggregation after resuspension, which provoked the measured increase in particle size [43].

On the other hand, SN-proticle formulations about 1030 nm showed a decrease in mean particle size after resuspension and high polydispersity. This indicates that the secondary aggregates, which were formed during the titration process, partly disassembled (most probably during resuspension). In both cases, the loading efficiencies of the components were similar to values before lyophilization. This again indicated that the loading efficiencies are mostly dependent on the mass ratio, as already discussed.

Concerning a favoured depot effect, in vivo images of SN-proticle formulations showed a closer and more retarded distribution pattern of SN than the control solutions. After 3 hours, there was still a detectable amount of fluorescently labeled SN at the site of injection when a SN-proticle formulation was applied. At the same time, no signal could be recorded after the administration of labeled SN in solution (control). However, the observed depot effect (i.m.) was not as obvious as in one of our previous distribution study after subcutaneous (s.c.) injection of peanut allergen Ara h 2 [26]. The difference can be explained by the different application methods, as the injection volume of i.m. application to mouse is limited to 30 μl, whereas 100 μl can be applied at s.c. administration. Further, the concentration of labeled Ara h 2 was twofold higher than the applied SN concentration at present study and the sensitivity of the camera also decreases with increase of tissue thickness.

## Conclusion

To conclude, the integration of the protamine titration process into the assembling of proticles demonstrated that the size of the resulting particles can be adjusted without influencing the loading efficiencies of secretoneurin. The particle size was mainly influenced by the applied formation method, but SN loading was only depending on the mass ratio of the components. Up to 65% of deployed SN could be successfully assembled into the proticle matrix and a basic approach of a depot effect in vivo was observable. In addition, lyophilization of proticles was shown for the first time. Due to the possible modification of the physicochemical properties various pharmacokinetic aspects like initial dose and modified drug release can be considered for the application of SN-proticle formulations.

## Supporting Information

S1 Fig Zeta potential distribution profiles of measurements throughout titration process. (PDF)

S2 Fig Determination of particle dimensions from obtained AFM images. (PDF)
