# Supplementary figures and images for: Manufacturing of a Secretoneurin Drug Delivery System with Self-Assembled Protamine Nanoparticles by Titration
Source: PLoS One. 2016 Nov 9;11(11):e0164149. doi: 10.1371/journal.pone.0164149 (PMC5102448; doi:10.1371/journal.pone.0164149)

S1 Fig. Zeta potential distribution profiles of measurements throughout titration process.

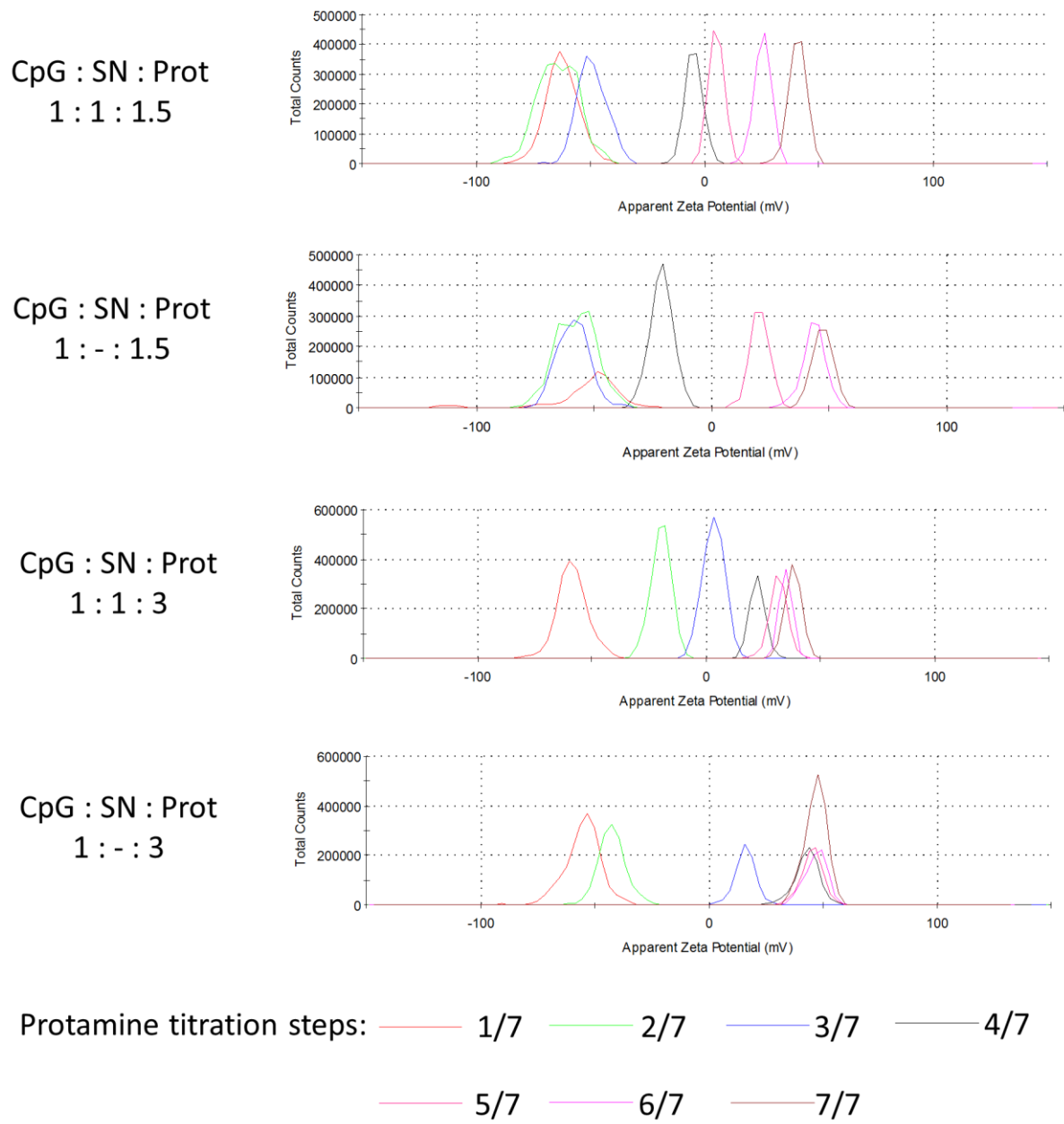

Supplement: S1 Fig — (PDF) [file pone.0164149.s001.pdf]
