# Supplementary material for: Manufacturing of a Secretoneurin Drug Delivery System with Self-Assembled Protamine Nanoparticles by Titration
Source: PLoS One. 2016 Nov 9;11(11):e0164149. doi: 10.1371/journal.pone.0164149 (PMC5102448; doi:10.1371/journal.pone.0164149)

S2 Fig. Determination of particle dimensions from obtained AFM images.

Particle dimensions were determined by using Imaris 7.3.0 (Bitplane, Zurich, Switzerland). AFM images were recorded for secretoneurin (SN) loaded particles with mass ratio of 1 : 1: 1.5 (ODN:SN:protamine) prepared by titration process (A) as well as by single protamine addition (B). Further, particles with mass ratio of 1 : 0.25 : 1.5 were determined (C).

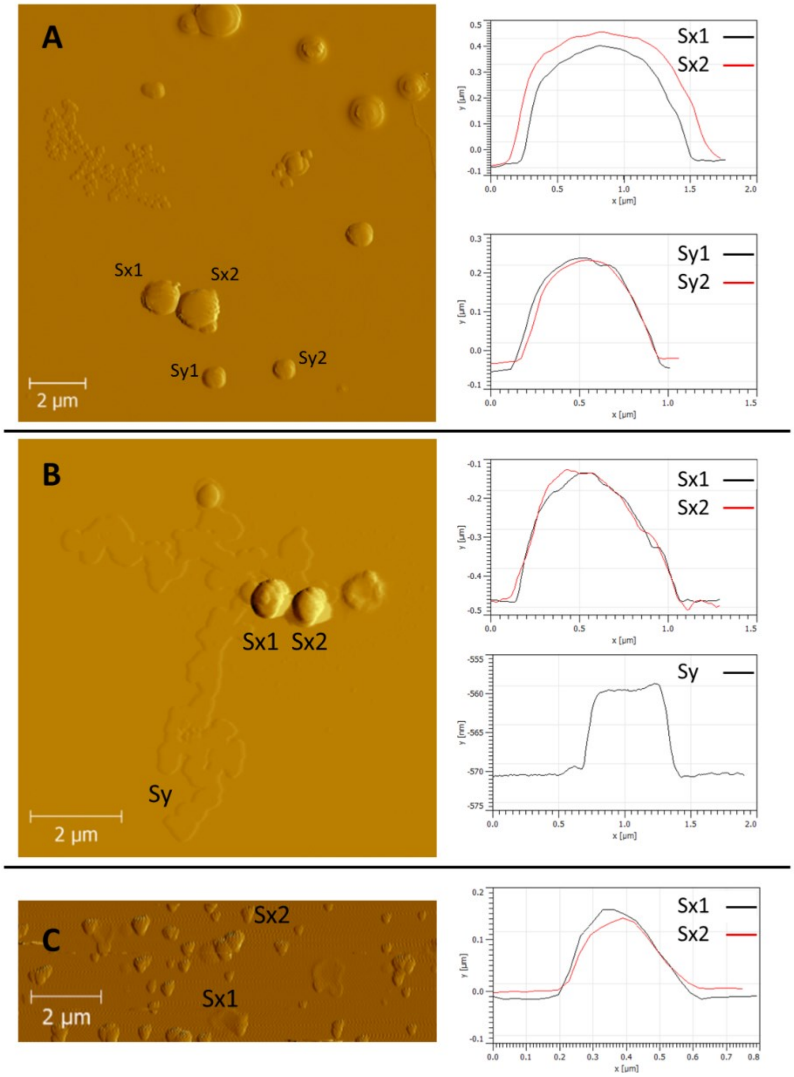

Supplement: S2 Fig — (PDF) [file pone.0164149.s002.pdf]
